# Associations between Aspects of Friendship Networks, Physical Activity, and Sedentary Behaviour among Adolescents

**DOI:** 10.1155/2014/632689

**Published:** 2014-09-24

**Authors:** Keri Jo Sawka, Gavin R. McCormack, Alberto Nettel-Aguirre, Anita Blackstaffe, Rosemary Perry, Penelope Hawe

**Affiliations:** ^1^University of Calgary, Teaching Research and Wellness Building, 3rd Floor, 3280 Hospital, Drive NW, AB, Canada T2N 4Z6; ^2^Alberta Children's Hospital, Room C4-435, 2888 Shaganappi, Trail NW, Calgary, AB, Canada T3B 6A8

## Abstract

*Background.* Adolescent friendships have been linked to physical activity levels; however, network characteristics have not been broadly examined. *Method.* In a cross-sectional analysis of 1061 adolescents (11–15 years), achieving 60 minutes/day of moderate-to-vigorous physical activity (MVPA) and participating in over 2 hours/day of sedentary behaviour were determined based on friendship network characteristics (density; proportion of active/sedentary friends; betweenness centrality; popularity; clique membership) and perceived social support. *Results.* Adolescents with no friendship nominations participated in less MVPA. For boys and girls, a ten percent point increase in active friends was positively associated with achievement of 60 minutes/day of MVPA (OR 1.11; 95% CI 1.02–1.21, OR 1.14; 95% CI 1.02–1.27, resp.). For boys, higher social support from friends was negatively associated with achieving 60 minutes/day of MVPA (OR 0.63; 95% CI 0.42–0.96). Compared with low density networks, boys in higher density networks were more likely to participate in over 2 hours/day of sedentary behaviour (OR 2.93; 95% CI 1.32–6.49). Social support from friends also modified associations between network characteristics and MVPA and sedentary behaviour. *Conclusion.* Different network characteristics appeared to have different consequences. The proportion of active close friends was associated with MVPA, while network density was associated with sedentary behaviour. This poses challenges for intervention design.

## 1. Introduction

Low levels of physical activity and high amounts of sedentary behaviour are two significant correlates of child and adolescent overweight and obesity [1]. Paediatric obesity is associated with cardiovascular risk factors such as high blood pressure, dyslipidemia, and insulin resistance [2]. On the other hand, regular participation in physical activity can improve bone mineral density, cardiorespiratory fitness, and body composition and reduce the risk of depression [3–6]. To accrue optimal health benefits, current Canadian physical activity guidelines recommend that adolescents accumulate at least 60 minutes of moderate-to-vigorous intensity physical activity (MVPA) every day [4] and limit their recreational screen time (i.e., sedentary behaviour) to 2 hours per day or less [3]. The guidelines also recommend that adolescents limit the amount of time they spend sitting for long periods and undertaking passive transportation [3]. Despite both adolescents and parents being aware of the potential health benefits [7], only 4% of girls and 9% of boys in Canada accumulate 60 minutes of MVPA on at least six days a week [8], and 60% of youth spend more than 2 hours per day participating in screen-based activities [9]. Thus, identifying modifiable determinants of physical activity and sedentary behaviour among adolescents is important for designing effective interventions.

Social contacts and personal relationships are important determinants of adolescent health and health behaviour, including physical activity [10–12]. Adolescents' immediate social environment includes, among other factors, relationships with parents, neighbours, friends, peers, teachers, and coaches [13]. These relationships can assist in the transfer, encouragement, and discouragement of adolescent attitudes and behaviour as well as contribute to adolescents' social and emotional wellbeing. Friends have a particular influence on adolescent health as a large portion of time is spent at school and participating in extracurricular activities. Mechanisms by which friends can influence individual behaviour include coparticipation (i.e., participating in the same behaviour with a friend), modeling (i.e., witnessing a friend or peer performing a behaviour), and social norms (i.e., perception of the amount the behaviour is performed by others or perception of approval of a behaviour) [14–16]. Qualitative evidence indicates that friends provide support for physical activity initiation via coparticipation, being an active model, and verbal support [17].

Recent studies have begun to use social network analysis to gain insight into the complex world of adolescent networks of friends and peers and their influence on adolescent physical activity [10, 11]. Social network analysis is both a theoretical paradigm and methodological tool that provides a means of quantifying relationships among entities, such as individuals or organizations, and estimating patterns of association [18]. Friendship nominations amongst a group of individuals can be aggregated to form quantitative estimates of an individual's friendship network. Analysis of friendship networks has been used to examine and explain adolescent health behaviour such as smoking [19], body mass index, and dietary behaviour [20], and more recently physical activity and sedentary behaviour [10, 11]. There is evidence to suggest associations between popularity, friend behaviour, and friendship reciprocity with regard to an individual's physical activity level; however, there are still mixed results on the association between aspects of friendship networks and sedentary behaviour [10, 11]. Gender differences also exist with regard to the influence of social networks on physical activity. For instance, friends' physical activity has been associated with boys', but not girls', physical activity [21–23].

Within social network analysis there are a variety of measures that examine individual positioning and relationships which have yet to be explored within the context of physical activity and sedentary behaviour but that have been examined in relation to other behaviours [24, 25]. These measures include ego-level variables such as clique membership (i.e., a group of at least three individuals who are all connected), betweenness centrality (i.e., an individual's tendency to link other network members), as well as network-level variables such as network density (i.e., number of connections in a network as a percentage of the total possible connections) [18]. These network measures could provide an additional layer of understanding and greater insight into the overall influence of friends on individual behaviour. Network density has been examined in the adolescent substance abuse literature [24, 25], and its applicability to physical activity and sedentary behaviour is worth investigating. While the associations between popularity and physical activity and sedentary behaviour have been examined [26–30], there has been less focus on those adolescents who receive no friendship nominations. Having fewer or no friends may result in limited opportunities for coparticipation in physical activity.

The aim of this study was twofold: (1) to examine the associations between aspects of school-based friendship networks (i.e., friendship network density, friend behaviour, popularity, and network roles), general perceived social support from friends, and achievement of recommended levels of physical activity and sedentary behaviour for adolescent boys and girls and (2) to examine the extent to which general perceived social support from friends modifies associations between friendship network measures and physical activity and sedentary behaviour.

## 2. Material and Methods

### 2.1. Data Source

This study is part of a larger Calgary-based (Alberta, Canada) project entitled Creating Opportunities for Resilience and Engagement (CORE) Connections. Six Catholic schools in Calgary were invited to participate in this study. The sample constituted all grades from 5 to 9 schools in the school district who had over 600 students in size and who did not offer specialized programming (e.g., performing arts, hockey). Seven schools were approached to take part and one refused. The schools were situated in different neighbourhoods within the metropolitan area. The median income of neighbourhoods in which the schools were situated ranged from $72,170 (School C) to $92,453 (School D) [31]—higher than the median income for all Calgary neighbourhoods ($67,238) [31]. In each school, all adolescents in grades from 7 to 9 were invited to participate in this study. A study information package was sent to homes seeking parental consent for their adolescent's participation. Two surveys were administered in school in November and December 2010. One survey captured physical activity, sedentary behaviour, general perceived social support from friends, and sociodemographic characteristics. The second survey captured adolescents' within-grade friendship network. Students completed online surveys on banks of computers sitting at least one meter apart. Research assistants explained the importance of privacy and confidentiality and monitored the room constantly, making sure that students were not looking at each other's screens.

### 2.2. Study Variables

#### 2.2.1. Physical Activity

Two survey items captured the number of days adolescents achieved at least 60 minutes of MVPA, outside of school hours, during (1) the past 7 days and (2) in a usual week [32]. The composite score, estimated from averaging responses to these two items, has acceptable test-retest reliability [32]. To reflect the current Canadian youth physical activity recommendations [4], we dichotomized the composite score into (1) achieving at least 60 minutes of MVPA on six or fewer days per week (*insufficiently active*) versus (2) achieving at least 60 minutes of MVPA on 7 days per week (*sufficiently active*).

#### 2.2.2. Sedentary Behaviour

Two survey items captured the time adolescents spend watching television or videos, using a computer, playing video games, or using a handheld device, outside of school, on a typical (1) weekday and (2) weekend day [33]. The average hours spent sedentary per day was estimated ([5 × weekday hours + 2 × weekend hours]/7 days per week) and dichotomized into (1) more than 2 hours per day (*high sedentary*) versus (2) 2 hours or less per day (*low sedentary*), reflecting the current Canadian adolescent sedentary behaviour guidelines [3]. Acceptable test-retest reliability for weekday and weekend television/video use (*r* = 0.80 and 0.69, resp.,) and weekday and weekend computer use (*r* = 0.66 and 0.71, resp.,) has been reported [33].

#### 2.2.3. General Perceived Social Support from Friends

General perceived social support from friends was measured through a social support scale consisting of four items. These items asked adolescents to report on how often (never, sometimes, most of the time, or all the time) they had friends who tried to help them; they could count on when things go wrong; they could share happy and sad times; and they could talk to about problems [34]. Responses to the four items were averaged, with higher scores reflecting higher levels of support. Internal consistency for this scale was acceptable (Cronbach's α = 0.82).

#### 2.2.4. Sociodemographic Characteristics

Socioeconomic status was measured using the family affluence scale (FAS) [35] which included four items asking adolescents to report the number of cars, vans, or trucks their family owned (i.e., 0, 1, or ≥2 vehicles); if they had their own bedroom (i.e., no = 0 or yes = 1); the number of times their family travelled away on holiday (i.e., 0, 1, 2, or ≥2 times in past 12 months); and the number of computers/laptops their family owned (i.e., 0, 1, 2, or ≥2). The responses to FAS items were summed and tertiled into low (FAS: <6), medium (FAS: 6-7), and high family affluence (FAS: ≥8). The FAS reflects household material wealth and it has been used in several studies investigating associations between family affluence, physical activity, and sedentary behaviour [36, 37], as well as in the Health Behaviour in School-Aged Children WHO Collaborative Cross-National Study [35]. Adolescents reported their gender, age (i.e., ≤12 years, 13 years, and ≥14 years old), how long they have lived in Canada (i.e., ≤5 years or >5 years), and residential relocation (i.e., did not move or moved at least once in the last 12 months). The school attended by the adolescent was also recorded (i.e., school A to F). The school attended may provide a proxy for community situatedness and affluence that was not captured in the surveys but could be important with regard to physical activity and sedentary behaviour.

#### 2.2.5. Social Network Variables

Adolescents were presented with a list of all individuals enrolled in their grade and were asked to indicate their closest friends. In other words, we used complete network survey methods, as opposed to ego-network methods, which ask respondents to name a certain number of friends and investigate the relationships among only those nominated. Using social network analysis software UCINET [38], seven ego-network variables were estimated based on the received (“incoming”) friendship nominations from close friends. The variables included (1) ego friendship network density (density); (2) proportion of received nominations from adolescents who achieved recommended levels of physical activity (proportion of active close friends); (3) proportion of received nominations from adolescents who participated in more than the recommended amount of sedentary behaviour (proportion of sedentary close friends); (4) amount of times an individual lies on the shortest path between two other individuals (betweenness centrality); (5) total number of nominations an adolescent received from other adolescents (popularity); (6) whether an adolescent has connections with at least two other adolescents and all three adolescents are connected through friendship nominations (clique member); and (7) if the adolescent received no friendship nominations. All variables were normalized using the number of adolescents in each grade. Density was dichotomized at the median density (12%) of the 18 networks (3 grades × 6 schools). Higher density reflects a higher connectivity between individuals within each grade. Clique member was dichotomized (i.e., not a member or member). All other social network variables were analyzed as numerical or continuous. The variables that were chosen—density, proportion of active close friends, proportion of sedentary close friends, popularity, clique member, and popularity—were consistent with theories of contagion in networks, theories of homophily (like people hanging out with like people), and balance theory (ties among triads), consistent with our interest in the generation and maintenance of norms, albeit that these were cross-sectional investigations [39]. We included betweenness centrality as it is a measure of social status and potential influence, due to the capacity to control flows of information [24]. It is only possible to measure in complete network surveys.

### 2.3. Statistical Analysis

Gender-stratified descriptive statistics including mean and standard deviations (SD) for numerical variables (i.e., general perceived social support from friends, proportion of active close friends, proportion of sedentary close friends, betweenness centrality, and popularity) and frequencies for categorical variables (i.e., age, FAS, school, time living in Canada, and residential relocation in the last 12 months, friendship network density, clique member, receiving no friendship nominations) were estimated. Gender-stratified independent samples* t*-tests, Pearson's chi-square tests, and subsequent* z*-tests for pairwise comparisons of proportions were undertaken to compare differences in all numerical and categorical variables, respectively.

Adjusted binary logistic regression models estimated the odds ratios (OR) and 95% confidence intervals (95% CI) for the association between sociodemographic variables (age, FAS, school attended, time living in Canada, and residential relocation), social network variables (friendship network density, proportion of active close friends, proportion of sedentary close friends, betweenness centrality, popularity, and clique member), and general perceived support from friends, and the likelihood of being (1) sufficiently active versus insufficiently active and (2) high sedentary versus low sedentary. Taking an exploratory approach, we also conducted a backward stepwise likelihood ratio test to identify significant interaction terms (*P* < 0.05) between general perceived social support from friends and each of the social network variables.

To aid in interpretation of the regression results, the proportion of active close friends and proportion of sedentary close friends were converted to percentages and rescaled so that a one-unit change was equal to a ten percentual point change in these variables. Adolescents who did not receive a friendship nomination were excluded from the regression models because at least one nomination was required for the calculation of the proportion of active close friends and proportion of sedentary close friends. Instead, Mann-Whitney* U* Tests were used to compare the amount of weekly physical activity and daily sedentary behaviour undertaken between those who did not receive a friendship nomination and those who received at least one friendship nomination. All analysis was conducted using SPSS version 20 [40].

## 3. Results and Discussion

From the six schools, all adolescents (*n* = 1,393) in grades 7 through 9 were invited to participate, of which 1,122 provided active consent (80.5%). A total of 1,061 adolescents subsequently provided complete data and were included in the analysis (76.2% of all those eligible).

### 3.1. Descriptive Statistics

The sample included 535 girls (50.4%) and 526 boys (49.6%), excluding adolescents who did not receive any close friendship nominations (Tables 1 and 2). Adolescents' age ranged from 11 to 15 years and was distributed as follows: 12 years and younger (boys = 40.9%, girls = 40.0%), 13 years (boys = 31.0%, girls = 33.3%), and 14 years and older (boys = 28.1%, girls = 26.7%). Similar percentages of boys and girls had high family affluence (boys = 37.5%, girls = 38.7%), middle family affluence (boys = 43.0%, girls = 44.5%), and low family affluence (boys = 19.6%, girls = 16.8%). A higher percentage of boys achieved recommended levels of physical activity per week compared with girls (boys = 16.0% and girls = 7.3%), while participation in at least two hours of sedentary activity per day was similar between boys and girls (boys = 79.8% and girls = 78.7%).

The mean number of incoming closest friend nominations for boys was 6.99 (SD = 3.79) and for girls was 6.52 (SD = 3.45). Network densities for close friendships across the schools and grades ranged from 7.0% to 14.0%. There were 21 adolescents (9 boys, 12 girls) who did not receive any friendship nominations. Among these adolescents, 7 (33.3%) were ≤12 years, 8 (38.1%) were 13 years, and 6 (28.6%) were ≥14 years of age. Moreover, 7 adolescents (33.3%) had low family affluence, 8 (38.1%) had medium family affluence, and 6 (28.6%) had high family affluence.

### 3.2. Physical Activity and Sedentary Behaviour among Those Who Received No Incoming Friendship Nominations

Adolescents who received no incoming nominations participated in significantly (*P* < 0.05) fewer days per week of at least 60 minutes of MVPA compared with those who received at least one friendship nomination (mean = 3.28 days/wk, SD = 1.76 days/wk versus 4.33 days/wk, SD = 1.81 days/wk, resp.). No difference in hours per day of sedentary behaviour was found.

### 3.3. Associations between Social Network-Derived Variables and Physical Activity and Sedentary Behaviour for Boys

Adjusting for all covariates, a ten percentage point increase in active close friends was significantly associated with an increased likelihood of being sufficiently active (OR 1.11; 95% CI 1.02–1.21) (Table 3). Boys with a higher general perceived social support from friends were significantly less likely to be sufficiently active (OR 0.63; 95% CI 0.42–0.96). Boys from school E were significantly less likely to be active compared with school A (OR 0.26; 95% CI 0.08–0.84). There were no significant interactions between social network variables and boys' general perceived social support from friends in relation to physical activity.

Adjusting for all covariates, boys in high density network were more likely to be highly sedentary compared with boys in low density networks (OR 2.93; 95% CI 1.32–6.49) (Table 3). Compared with boys ≤12 years of age, boys ≥14 years of age were more likely to be highly sedentary (OR 2.23; 95% CI 1.04–4.77). Moreover, boys in schools C (OR 2.92; 95% CI 1.04–8.21) and F (OR 4.24; 95% CI 1.30–13.77) were significantly more likely to be highly sedentary compared with boys in school A. There was a significant interaction (*P* < 0.05) between both the proportion of active close friends (OR 1.12; 95% CI 1.00–1.26) and proportion of sedentary close friends (OR 1.16; 95% CI 1.01–1.32) and general perceived social support from friends and being highly sedentary.

### 3.4. Associations between Social Network-Derived Variables and Physical Activity and Sedentary Behaviour for Girls

After adjusting for all covariates, a ten percentage point increase in active close friends was associated with achieving sufficient levels of physical activity (OR 1.14; 95% CI 1.02–1.27) (Table 4). No other covariates were significantly associated with sufficient levels of physical activity among girls. There was a significant interaction (*P* < 0.05) between the proportion of sedentary close friends and general perceived support from friends in relation to being sufficiently active (OR 1.31; 95% CI 1.04–1.67).

Adjusting for all other covariates, girls in schools C, E, and F were more likely to be highly sedentary (OR 2.89; 95% CI 1.22–6.83, OR 2.71; 95% CI 1.03–7.13, OR 6.18; 95% CI 1.94–19.64, resp.,) compared to girls in school A (Table 4). There was a significant interaction (*P* < 0.05) between general perceived social support from friends and clique membership and a decreased likelihood of being highly sedentary (OR 0.38; 95% CI 0.15–0.96). No other significant associations were found between the other covariates and sedentary behaviour among girls.

### 3.5. Discussion

The low prevalence of participation in at least 60 minutes of MVPA every day and high prevalence of participation in over 2 hours per day of sedentary behaviour (i.e., recreational screen time) in our sample of adolescents is consistent with other Canadian studies [9, 41]. For boys and girls, a higher proportion of active close friends were associated with an increased likelihood of achieving sufficient levels of physical activity. We also found that, for boys, friendship network density was positively associated with sedentary behaviour. An important finding was that adolescents who received no friendship nominations spent significantly fewer days per week participating in 60 minutes of MVPA compared with adolescents who received at least one friendship nomination. Our study highlights the potential importance of close friendship network characteristics in influencing physical activity and sedentary behaviour in adolescents.

The association between close friends' physical activity and an individual's physical activity was consistent with previous findings [10, 11]; however, studies that undertook gender-stratified analysis found associations for friends' physical activity and physical activity among boys, but not among girls [21–23]. Similar to Sirard et al. [42] who found that friend's weekly hours of MVPA were significantly associated with boy's and girl's physical activity, we found that, regardless of gender, a higher proportion of active close friends were positively associated with achieving sufficient levels of physical activity. Having close friends who are active appears beneficial; however, our findings also suggest that not being nominated as a close friend may have a negative impact on physical activity behaviour. While few adolescents did not receive any close friendship nominations (*n* = 21), they were found to participate in less MVPA than those who received at least one close friendship nomination. Similar results were found elsewhere regarding other health-related behaviour. For example, isolate adolescents are significantly more likely to smoke compared with adolescents who are socially connected to others (i.e., clique members) [19]. It is possible that adolescents who are not considered a close friend by others receive limited support or encouragement to participate in physical activity and may have no opportunities to coparticipate in physical activities with others. A complete network analysis, like that conducted here, allowed the investigators to observe those without friendship nominations, while an ego-network analysis, by definition, would not.

This study was able to contribute knowledge relating to network density and network positioning and physical activity and sedentary behaviour. Boys who were in a higher density network were more likely to be sedentary compared with those in a low density network. As the majority of boys were sedentary (80%), a higher density network may have allowed for more exposure to normative attitudes, ideals, and behaviour among adolescents within the network, which could result in an increased likelihood of an individual being highly sedentary. Haynie [43] found similar results for adolescent delinquency; the interaction between high density and delinquent peer networks resulted in higher delinquency involvement.

Although not the primary focus of our study, there were significant differences in the likelihood of adolescents being sufficiently active, as well as highly sedentary, among the six schools. This study, which is part of a larger project focused on improving health and wellbeing, was not initially designed to capture information about school policies, programs, or opportunities for physical activity and sedentary behaviour. Speculatively, it is possible that school characteristics and opportunities may have contributed to differences in physical activity and sedentary behaviour. For example, a review by Bonell et al. [44] found that schools with higher attainment and attendance, combined with lower truancy, had lower rates of student substance abuse. With regard to physical activity, Cradock et al. [45] found that characteristics of school campuses (i.e., school campus area per student, building area per student, and play area per student) were each associated with higher levels of physical activity. Therefore, it is possible that school characteristics may have accounted for the differences in levels of sufficiently active and highly sedentary adolescents among the schools in this study.

Evidence indicates that perceived social support for physical activity is positively associated with physical activity among adolescents [12, 46, 47]. Our study did not measure perceived support for physical activity but assessed the role of feeling emotionally supported by friends, which was not limited to within-school friends. We found evidence of effect modification between perceived social support and friendship network variables in relation to sedentary behaviour. Boys who reported higher general perceived social support from friends and had a higher proportion of* active* close friends were more likely to be sedentary, and boys who reported higher general perceived social support from friends and had a higher proportion of* sedentary* close friends were also more likely to be sedentary. We are unaware of previous studies examining the interaction between perceived social support from friends and social network variables in relation to physical activity and sedentary behaviour. Reasons for these counter-intuitive findings are therefore speculative. The findings could reflect patterns of social interaction among adolescents that may provide individuals with a virtual (e.g., Facebook, online gaming) versus physical form (e.g., sports teams, face-to-face games) of social support. Boys may also receive social support from friends who are both sufficiently active and highly sedentary; boys may participate in team sports with friends and also participate in sedentary activities with these friends, such as watching televised professional sports together. Future research may wish to examine the extent to which “influential” friends within social networks influence physical activity and sedentary behaviour, as well as the role of peer pressure, starting with qualitative studies.

Interactions between general perceived social support and social network variables were also found for girls. Girls who reported higher perceived general social support from friends and had a higher number of sedentary close friends were more likely to be sufficiently active. Moreover, girls who reported higher perceived social support from friends and who were members of a clique were less likely to be highly sedentary. It is possible that girls may have nonschool-based friends with whom they are active (e.g., sports teams). Our analysis did not examine the gender distribution of individuals' friendship networks. Girls may receive social support from their female friends, who also happen to be highly sedentary but also have male friends with whom they participate in physical activities. Some of our counter-intuitive findings for boys and girls may reflect our measure of general perceived social support which did not capture social support specifically associated with physical activity or sedentary behaviour. There may also be extenuating factors, such as family support, which have been shown to influence adolescent physical activity [48] and sedentary behaviour [49] that were not accounted for in this analysis.

This study has several limitations that should be considered. Causal inferences cannot be drawn from this cross-sectional study. Previous longitudinal analyses have shown that friends' physical activity tends to become similar over time [21, 27, 50–52], indicating a process of friendship influence or socialization; however, several of these studies [50–52] also found that adolescents tend to select their friends based on similarities in physical activity. The low prevalence of sufficiently active boys (16.0%) and girls (7.3%) may have limited the statistical power to detect some meaningful associations from the regression models. The mismatch in context between our physical activity and sedentary behaviour measures (i.e., behaviour both inside and outside of school) and social network measure (i.e., close friends inside the school) might have resulted in fewer significant associations being found.

## 4. Conclusions

The determinants of physical activity and sedentary behaviour in children and adolescents are multifaceted and complex. Individual-level behaviour (e.g., motor ability and skill) and psychological characteristics (e.g., self-efficacy, attitudes, enjoyment), the social environment (e.g., friends and parents, family relationships and structure, and culture), the physical environment (e.g., neighbourhood parks, play equipment, availability and access to screen-based devices, and urban design), policy (e.g., mandatory physical education and activity breaks in schools), and programs (e.g., walking school bus) together influence patterns of physical activity and sedentary behaviour that adolescents undertake [48]. Our study focused on the influence of the social environment only and more specifically one aspect of the social environment—adolescent school-based friendship networks—on physical activity and sedentary behaviour. Our study findings suggest that characteristics of school-based close friendship networks are differentially associated with physical activity and sedentary behaviour. Specifically, social network-derived variables associated with physical activity differ from those associated with sedentary behaviour; relationships between individuals' and the proportion of active close friends appear to be associated with physical activity, while network density appears to be associated with sedentary behaviour.

Results from this study invite consideration of future public health interventions which utilize friendship influence to increase physical activity among adolescents. While we recognize that close friendship is a matter of complex personal choice, the opportunities to get to know new and more people, which could lead to this choice, are possibly amenable. This requires further investigation and understanding. Increasing the proportion of active individuals within a friendship network, particularly those with a higher number of friends, may result in a snowball effect and increase the likelihood of other individuals becoming sufficiently active. Harnessing the benefits of positive friendship influence to promote modeling and coparticipation could help adolescents achieve the recommended levels of physical activity required for optimal health benefits. In other fields, attempts have been made to harness the properties of friendship networks to improve health behaviour [53]. However, we have a partial view of a more complicated picture. Not only can an individual be both sedentary and active [54] we have shown that different aspects of networks appear to support these behaviour differently. The low prevalence of sufficiently active and high prevalence highly sedentary adolescents in our study is worrying and therefore suggests that broader multifaceted community, environmental, and school-based interventions may be of more immediate practical benefit [55, 56].

## Figures and Tables

**Table 1:** Descriptive statistics for the sociodemographic characteristics, general perceived social support from friends, physical activity, and sedentary behaviour for boys (*n* = 526).

	Physical activity		Sedentary behaviour	
	Sufficiently active (≥60 min of MVPA every day)	Insufficiently active (≥60 min of MVPA on <7 days/week)	High sedentary (>2 hrs/day of sedentary behaviour)	Low sedentary (≤2 hrs/day of sedentary behaviour)
Sociodemographic characteristics				
Age [*n* (%)]				
12 years and younger	37 (17.2)	178 (82.8)	157 (73.0)	58 (27.0)^b^
13 years	31 (19.0)	132 (81.0)	132 (81.0)	31 (19.0)
14 years and older	16 (10.8)	132 (89.2)	132 (89.2)	16 (10.8)^b^
Family affluence [*n* (%)]				
Low	11 (10.7)	92 (89.3)^a^	80 (77.7)	23 (22.3)
Medium	32 (14.2)	194 (85.8)	177 (78.3)	49 (21.7)
High	41 (20.8)	156 (79.2)^a^	164 (83.2)	33 (16.8)
Length of time in Canada [*n* (%)]				
More than 5 years	6 (9.0)	61 (91.0)	56 (83.6)	11 (16.4)
5 years or less	78 (17.0)	381 (83.0)	365 (79.5)	94 (20.5)
Number of times moved last year [*n* (%)]				
Did not move	74 (17.2)	356 (82.8)	340 (79.1)	90 (20.9)
Moved at least once	10 (10.4)	86 (89.6)	81 (84.4)	15 (15.6)
Social network characteristics				
Incoming close friend nominations [*n* (%)]				
Received ≥1 nomination	87 (16.1)	453 (83.9)	432 (80.0)	108 (20.0)
Received no nominations	0 (0.0)	9 (100.0)	5 (55.6)	4 (44.4)
Proportion active close friends [mean (SD)]	0.40 (0.4)^c^	0.30 (0.3)^c^	0.30 (0.3)^d^	0.38 (0.3)^d^
Proportion sedentary close friends [mean (SD)]	0.67 (0.3)	0.72 (0.3)	0.72 (0.3)	0.68 (0.3)
Betweenness centrality [mean (SD)]	3.63 (4.2)	3.07 (4.2)	3.14 (4.3)	3.26 (3.9)
Popularity (incoming nominations) [mean (SD)]	7.08 (3.6)	6.98 (3.8)	6.89 (3.7)	7.40 (4.0)
Clique member [*n* (%)]				
Not a member	35 (18.0)	155 (82.0)	156 (82.1)	34 (17.9)
Member	49 (14.6)	287 (85.4)	265 (78.9)	71 (21.1)
Perceived support from friends [mean (SD)]^f^	3.15 (0.7)	3.28 (0.6)	3.27 (0.6)	3.20 (0.6)
Total boys [*n* (%)]	84 (16.0)	442 (84.0)	421 (80.0)	105 (20.0)

^a,b^Significant (*P* < 0.05) chi-square and Bonferroni-adjusted pair-wise comparison (*z*-test); ^c,d^significant (*P* < 0.05) difference in means (Mann-Whitney *U*-test), ^f^average general perceived social support index: 1 = received support none of the time to 4 = received support all of the time in increments of 0.25, MVPA: moderate-to-vigorous intensity physical activity, SD: standard deviation.

**Table 2:** Descriptive statistics for the sociodemographic characteristics, general perceived social support from friends, physical activity, and sedentary behaviour for girls (*n* = 535).

	Physical activity		Sedentary behaviour	
	Sufficiently active (≥60 min of MVPA every day)	Insufficiently active (≥60 min of MVPA on <7 days/week)	High sedentary (>2 hrs/day of sedentary behaviour)	Low sedentary (≤2 hrs/day of sedentary behaviour)
Sociodemographic characteristics				
Age [*n* (%)]				
12 years and younger	22 (10.3)	192 (89.7)	156 (72.9)	58 (27.1)^a^
13 years	11 (6.2)	167 (93.8)	140 (78.7)	38 (21.3)
14 years and older	6 (4.2)	137 (95.8)	122 (85.3)	21 (14.7)^a^
Family affluence [*n* (%)]				
Low	6 (6.7)	84 (93.3)	70 (77.8)	20 (22.2)
Medium	14 (5.9)	224 (94.1)	186 (78.2)	52 (21.8)
High	19 (9.2)	188 (90.8)	162 (78.3)	45 (21.7)
Length of time in Canada [*n* (%)]				
More than 5 years	3 (5.3)	54 (94.7)	43 (75.4)	14 (24.6)
5 years or less	36 (7.5)	442 (92.5)	375 (78.5)	103 (21.5)
Number of times moved last year [*n* (%)]				
Did not move	28 (6.6)	397 (93.4)	336 (79.1)	89 (20.9)
Moved at least once	11 (10.0)	99 (90.0)	82 (74.5)	28 (25.5)
Social network characteristics				
Incoming close friend nominations [*n* (%)]				
Received ≥1 nomination	39 (7.2)	503 (92.8)	425 (78.4)	117 (21.6)
Received no nominations	0 (0.0)	12 (100.0)	11 (91.7)	1 (8.3)
Proportion active close friends [mean (SD)]	0.43 (0.4)^c^	0.25 (0.4)^c^	0.24 (0.3)^d^	0.34 (0.4)^d^
Proportion sedentary close friends [mean (SD)]	0.75 (0.3)	0.74 (0.3)	0.76 (0.3)	0.70 (0.3)
Betweenness centrality [mean (SD)]	2.68 (3.0)	3.16 (4.0)	3.26 (4.0)	2.66 (3.5)
Popularity (incoming nominations) [mean (SD)]	6.36 (3.1)	6.53 (3.5)	6.67 (3.6)^e^	5.97 (2.7)^e^
Clique member [*n* (%)]				
Not a member	9 (6.1)	138 (93.9)	115 (78.2)	32 (21.8)
Member	30 (7.7)	358 (92.3)	303 (78.1)	85 (21.9)
Perceived support from friends [mean (SD)]^f^	3.54 (0.6)	3.53 (0.5)	3.53 (0.5)	3.53 (0.5)
Total girls [*n* (%)]	39 (7.3)	496 (92.7)	418 (78.1)	117 (21.9)

^a,b^Significant (*P* < 0.05) chi-square and Bonferroni-adjusted pair-wise comparison (*z*-test); ^c,d,e^significant (*P* < 0.05) difference in means (Mann-Whitney *U* test), ^f^average general perceived social support index: 1 = received support none of the time to 4 = received support all of the time in increments of 0.25, MVPA: moderate-to-vigorous intensity physical activity, SD: standard deviation.

**Table 3:** Odds ratios (OR) and 95% confidence intervals (95% CI) for the association between sociodemographic characteristics, social network variables, general perceived social support from friends, physical activity, and sedentary behaviour for boys (*n* = 526).

	Sufficiently active (≥60 min of MVPA every day)	High sedentary (>2 hrs/day of sedentary behaviour)	
	Adjusted main effects OR (95% CI)	Adjusted main effects OR (95% CI)	Adjusted main effects and interaction OR (95% CI)
**Sociodemographic characteristics**			
School			
A^#^	1.00	1.00	1.00
B	0.43 (0.18–1.06)	2.34 (0.95–5.74)	2.40 (0.90–6.01)
C	0.42 (0.15–1.15)	2.92 (1.04–8.21)∗	2.94 (1.02–8.47)∗
D	0.67 (0.22–2.06)	1.54 (0.50–4.77)	1.50 (0.48–4.74)
E	0.26 (0.08–0.84)∗	1.95 (0.66–5.76)	1.76 (0.58–5.32)
F	0.51 (0.16–1.61)	4.24 (1.30–13.77)∗	4.00 (1.20–13.33)∗
Age			
12 yrs and younger^#^	1.00	1.00	1.00
13 yrs	1.40 (0.71–2.75)	1.09 (0.58–2.08)	1.16 (0.61–2.22)
14 yrs and older	0.83 (0.35–1.95)	2.23 (1.04–4.77)∗	2.39 (1.10–5.18)∗
Family affluence			
Low^#^	1.00	1.00	1.00
Middle	1.29 (0.60–2.77)	1.21 (0.66–2.23)	1.25 (0.68–2.31)
High	2.00 (0.94–4.27)	1.55 (0.81–2.94)	1.54 (0.80–2.94)
Length of time in Canada			
More than 5 years^#^	1.00	1.00	1.00
5 years or less	0.48 (0.19–1.25)	1.23 (0.58–2.63)	1.25 (0.58–2.70)
Number of times moved last year			
Did not move^#^	1.00	1.00	1.00
Moved at least once	0.75 (0.35–1.61)	1.10 (0.58–2.10)	1.01 (0.52–1.94)
**Social network characteristics**			
Density			
Low (density <12%)^#^	1.00	1.00	1.00
High (density ≥12%)	0.56 (0.23–1.33)	2.93 (1.32–6.49)∗	2.99 (1.34–6.69)∗
Proportion of active close friends	1.11 (1.02–1.21)∗	0.96 (0.89–1.03)	0.66 (0.00–0.96)∗
Proportion of sedentary close friends	1.02 (0.92–1.12)	0.91 (0.83–1.01)	0.58 (0.00–0.88)∗
Betweenness centrality	1.02 (0.96–1.08)	1.00 (0.95–1.07)	1.01 (0.95–1.07)
Popularity	1.02 (0.97–1.07)	0.98 (0.93–1.03)	0.97 (0.93–1.02)
Clique member			
Member^#^	1.00	1.00	1.00
Not a member	1.21 (0.68–2.16)	1.32 (0.76–2.27)	1.31 (0.75–2.27)
**General perceived social support from friends** ^ a^	0.63 (0.42–0.96)∗	1.35 (0.92–1.98)	0.34 (0.12–1.03)

**Interactions**			
Proportion of active close friends ∗ General perceived social support from friends			1.12 (1.00–1.26)∗
Proportion of sedentary close friends ∗ General perceived social support from friends			1.16 (1.01–1.32)∗

*P < 0.05, ^#^referent category, ^a^average general perceived social support index: 1 = received support none of the time to 4 = received support all of the time in increments of 0.25, MVPA: moderate-to-vigorous physical activity, OR: odds ratio, CI: confidence interval.

No significant interactions between friendship network characteristics and general perceived social support for boys' physical activity.

**Table 4:** Odds ratios (OR) and 95% confidence intervals (95% CI) for the association between sociodemographic characteristics, social network variables, general perceived social support from friends, physical activity, and sedentary behaviour for girls (*n* = 535).

	Sufficiently active (≥60 min of MVPA every day)		High sedentary (>2 hrs/day of sedentary behaviour)	
	Adjusted main effects OR (95% CI)	Adjusted main effects and interaction OR (95% CI)	Adjusted main effects OR (95% CI)	Adjusted main effects and interaction OR (95% CI)
Sociodemographic characteristics				
School				
A^#^	1.00	1.00	1.00	1.00
B	0.17 (0.03–1.04)	0.16 (0.03–1.00)	1.73 (0.78–3.88)	1.79 (0.80–4.03)
C	0.86 (0.24–3.15)	0.81 (0.22–2.94)	2.89 (1.22–6.83)∗	3.10 (1.30–7.38)∗
D	0.83 (0.16–4.24)	0.90 (0.17–4.66)	1.21 (0.46–3.18)	1.20 (0.46–3.16)
E	0.38 (0.07–2.01)	0.38 (0.07–2.07)	2.71 (1.03–7.13)∗	2.85 (1.08–7.52)∗
F	0.96 (0.19–4.73)	0.99 (0.20–4.89)	6.18 (1.94–19.64)∗	6.87 (2.11–22.35)∗
Age				
12 yrs and younger^#^	1.00	1.00	1.00	1.00
13 yrs	0.68 (0.27–1.74)	0.69 (0.27–1.79)	1.19 (0.66–2.17)	1.16 (0.64–2.12)
14 yrs and older	0.50 (0.14–1.80)	0.48 (0.13–1.77)	1.61 (0.78–3.35)	1.63 (0.78–3.41)
Family affluence				
Low^#^	1.00	1.00	1.00	1.00
Middle	0.88 (0.31–2.49)	0.79 (0.28–2.27)	1.11 (0.60–2.07)	1.09 (0.58–2.04)
High	1.46 (0.53–4.03)	1.41 (0.51–3.92)	1.15 (0.61–2.18)	1.16 (0.61–2.21)
Length of time in Canada				
More than 5 years^#^	1.00	1.00	1.00	1.00
5 years or less	0.54 (0.15–2.01)	0.61 (0.16–2.28)	0.76 (0.37–1.55)	0.76 (0.37–1.56)
Number of times moved last year				
Did not move^#^	1.00	1.00	1.00	1.00
Moved at least once	1.55 (0.70–3.42)	1.60 (0.72–3.54)	0.82 (0.48–1.40)	0.91 (0.53–1.58)
Social network characteristics				
Density				
Low (density <12%)^#^	1.00	1.00	1.00	1.00
High (density ≥12%)	1.05 (0.30–3.65)	1.07 (0.30–3.81)	1.43 (0.70–2.94)	1.48 (0.72–3.04)
Proportion of active close friends	1.14 (1.02–1.27)∗	1.15 (1.03–1.28)∗	0.94 (0.88–1.01)	0.94 (0.88–1.00)
Proportion of sedentary close friends	1.02 (0.88–1.19)	0.00 (0.00–0.90)∗	0.96 (0.88–1.05)	0.96 (0.88–1.05)
Betweenness centrality	0.96 (0.87–1.07)	0.96 (0.86–1.06)	1.04 (0.97–1.11)	1.76 (0.96–3.22)
Popularity	0.99 (0.91–1.07)	0.98 (0.91–1.07)	1.03 (0.99–1.10)	1.03 (0.98–1.09)
Clique member				
Member^#^	1.00	1.00	1.00	1.00
Not a member	0.84 (0.34–2.04)	0.75 (0.30–1.86)	1.38 (0.80–2.41)	39.86 (1.54–1034.20)∗
General perceived social support from friends^a^	1.06 (0.55–2.04)	0.14 (0.02–0.88)	0.98 (0.65–1.48)	1.88 (0.99–3.55)

Interactions				
Proportion of sedentary close friends ∗ General perceived social support from friends		1.31 (1.04–1.67)∗		
Clique member ∗ General perceived social support from friends				0.38 (0.15–0.96)∗

*P < 0.05, ^#^referent category, ^a^average general perceived social support index: 1 = received support none of the time to 4 = received support all of the time in increments of 0.25, MVPA: moderate-to-vigorous physical activity, OR: odds ratio, CI: confidence interval.
